# Influence of Partnership Relationships on Long-Term Neurological Rehabilitation in Germany: Protocol for a Qualitative Retrospective Study

**DOI:** 10.2196/63949

**Published:** 2025-01-13

**Authors:** Alexa von Bosse, Peter König, Eva Jansen

**Affiliations:** 1 Institute of Medical Sociology and Rehabilitation Science Charité - Universitätsmedizin Berlin corporate member of Freie Universität Berlin and Humboldt-Universität zu Berlin Berlin Germany; 2 Faculty Health Safety Society Furtwangen University Furtwangen Germany

**Keywords:** neurological rehabilitation, neurological injury, therapeutic alliance, relationship building, caregivers, family, partnership, health professionals, neurological, therapeutic, Germany, retrospective study, narrative interview, biopsychosocial, family-centered

## Abstract

**Background:**

Acquired neurological diseases entail significant changes and influence the relationship between a patient and their significant other. In the context of long-term rehabilitation, those affected collaborate with health care professionals who are expected to have a positive impact on the lives of the affected individuals.

**Objective:**

This study aims to examine the changes in the relationship between the patient and their loved ones due to acquired neurological disorders and the influence of health care professionals on this relationship.

**Methods:**

Through sociogenetic type building, we will identify different types of patient-caregiver dyads and their effects on health care professionals and vice versa. The results will then be integrated into a model based on the theory of symbolic interactionism and Baxter’s Relational Dialectics Theory.

**Results:**

This study is not funded and was approved by the ethics committee of the German Society for Nursing Science, and it complies with the Declaration of Helsinki. The data collection started in June 2024 based on narrative couple interviews and is running. We assume that patients and their relatives will demonstrate heterogeneity as individuals, as well as in their interactions within the dyad, regarding certain orientations such as coping with illness, motivation for therapy, and coping strategies.

**Conclusions:**

Our findings address a biopsychosocial perspective that enhances treatment approaches in neurological long-term care. Understanding the influence of professionals on dyadic couple relationships can improve rehabilitation effectiveness by tailoring therapeutic approaches to various patient types, relatives, and dyadic relationship constellations. This fosters patient- and family-centered therapy in line with holistic care.

**International Registered Report Identifier (IRRID):**

DERR1-10.2196/63949

## Introduction

### Scientific and Practical Relevance of the Project

Rehabilitation is effective for any person with a long-lasting disability at any stage of the illness. A patient-centered biopsychosocial rehabilitation approach involving a multidisciplinary team is particularly essential for individuals with neurological conditions, as they frequently experience lifelong limitations and require comprehensive, individualized support to enhance their quality of life and achieve maximum independence [1].

Neurological damage is acquired through stroke, traumatic central nerve injury, or traumatic brain injury. The resulting sudden loss of independence often leads to irreversible harm and is one of the main causes of lifelong support and home care needs. Strokes cause a high economic and health burden [2]. For example, the cost of stroke to health care systems in 32 countries totaled US $28.2 billion in 2017 [3]. This is why the Stroke Alliance for Europe emphasizes the need for European countries to invest in cost-effective stroke interventions to improve rehabilitation and the quality of life of stroke survivors [3]. The number of patients with traumatic brain injury in 2016 in Germany was 419,507 [4]. More than 4000 patients become long-term care cases due to severe permanent injuries, resulting in high costs and limited ability to work [5]. The rehabilitation process for neurological diseases is highly complex, depends on numerous influencing factors, and is vague concerning the results. The effectiveness of rehabilitation is highly dependent on its integration within a biopsychosocial framework and requires an individualized, person-centered approach [1]. Functional, cognitive, and psychological challenges directly impact the ability to perform daily tasks independently [6]. Acquired neurological damage leads to a loss of social roles for those affected and requires adaptation to the new living conditions [7]. In the context of illnesses, the individual coping strategies used vary greatly across the different patients [8]. Life orientations, goals, ideas, and expectations about one’s own life and future development represent a relevant aspect of rehabilitative cooperation and success. Relatives of those who are directly affected not only experience trauma but also deal with the illness and its lasting personal consequences for themselves [9]. Individual processes take place within partnership dyads and domestic cohabitation. This is particularly relevant for Germany where 63% of people in need of care are cared for exclusively by relatives in a domestic setting. Nursing homes care for only 16% of people in need of long-term care in Germany [10].

### Family Change Processes and Relationship Dynamics

In the partnership dyad between the person affected and the caregivers, dual roles arise as a spouse, patient, or caring relative [11]. Changes caused by a sudden loss of independence and the need for assistance due to a family member’s illness impact power dynamics, role distributions, tasks, and decision-making between patients and their relatives. On the one hand, relatives provide their resources while on the other hand, they need help themselves. Within the framework of the existing family system, relatives thus have to face new challenges [9,12]. In this context, the rehabilitation process after acquired neurological damage is associated with fears, worries, and adaptations for many patients and their caregivers [13].

The therapeutic relationship significantly influences the rehabilitation process of neurological diseases and is of paramount importance [14,15].

Family caregivers play a central role in providing care, therapy, and social support, particularly in the case of lifelong disability due to acquired neurological damage [16]. According to recent international studies, the psychosocial effects of neurological damage have already been described for patients [12,17] and family caregivers of affected persons [18]. Studies also explore the dynamics between patients and therapists, as well as the relationships formed in patient-therapist interactions [12,19]. However, given the importance of biopsychosocial aspects in therapy and the close interactions affecting the well-being of patients and their relatives, there is a lack of studies on the influence of health care professionals on the dyadic relationship between patients and relatives in different phases of rehabilitation.

Our study addresses the following questions:

1. How does the dyadic relationship between persons with acquired central nervous chronic impairment and their significant others change over time and what does it mean for the therapeutic process?

2. How do relationships and relationship aspects influence the rehabilitation process?

3. What is the influence on the dyadic relationship that affects persons and their life partners which attributes to the health care professionals?

Furthermore, we want to discuss how health care professionals should react to the different situations and dyads in the rehabilitation process to enable the best possible quality of life from the perspective of the affected person and their life partner.

### Underpinning Theories and Theoretical Approach

The theory of symbolic interactionism (SI) is based on the fundamental idea that actors influence each other within the framework of interaction processes and the resulting ongoing interpretations [20]. In relation to the object of the study, this means that 2 people enter a relationship with each other based on a “self” resulting from their history of interaction. The “self” is called into question by the sudden injury that leads to a long-term need for help, is no longer connectable in the strongly changed interaction, and needs a challenging adaptation.

Building on the understanding of SI, Leslie Baxter’s Relational Dialectics Theory (RDT) focuses on forming a meaningful relationship between partners [21]. The relationship between the patient and the caregiver is established in dialogue and is subject to continuous change. Examples of dialectics within the dyadic relationship are integration-separation, stability-change, or expression-nonexpression. According to the RDT, these dialectics are natural and unavoidable in any relationship, and they play a crucial role in shaping how individuals communicate and interact [22]. This is particularly relevant to understand the changes within the couple relationship of patient and relative over time.

From a theoretical perspective, this study aims to increase the knowledge on the influence of changes in the couple’s relationship on the rehabilitation process and vice versa, and consequently on the quality of life of patients and caregivers. Different types of patient-caregiver dyads can be identified and empirically validated based on theoretical approaches. This approach makes it possible to link empirically identified types back to the theory of SI [20], and the RDT [21], to support them empirically or to change, refute, or expand them. A major novelty of the study lies in the theoretical foundation of the reciprocal effects of relational changes in acquired central nervous damage in rehabilitative processes and interaction with health care professionals.

From a practical perspective, standardized types of dyads can be recognized, and various rehabilitative approaches can be postulated at the practice level on a sociopsychological basis. This enables the enhancement of individual care for affected individuals, with a focus on improving their quality of life.

## Methods

The study follows the Standards for Reporting Qualitative Research [23].

### Approach, Sampling, and Recruitment

#### Approach

We use a qualitative approach, exploring how individuals maintain and shape their relationships in response to changed life situations within a domestic environment.

#### Sampling

Our sample consists of people with similar neurological conditions. Within the scope of the study, we include adult couples in which one person is chronically affected by an acquired neurological injury. Within these cases, disturbances, social status, age, and duration of affliction should be heterogeneous to ensure a high degree of diversity.

The inclusion criteria are (1) people who had an acquired central nervous system injury at least 6 months before and their partners, (2) the partnership existed before the injury and still exists at the time of the survey, (3) partners live together in a household, (4) participants have the capacity to give consent, (5) participants exhibit sufficient visual and hearing ability in order to understand the questions during the interview, (6) residence in Germany, and (7) sufficient understanding of German language.

#### Recruitment and Field Access

We recruit participants by displaying notices and flyers in public areas, including doctor’s waiting rooms and neurological rehabilitation centers. This approach promotes voluntary participation. The study aims to achieve theoretical saturation without relying on predetermined statistical methods. Participants and their partners may choose the location for the interviews, which can be either through video call or in a place of their preference. The decision regarding the interview setting (face-to-face or online) is made by the concerned family dyads to avoid complicating the caregiving situation. No payment or reimbursement of expenses is provided for participation.

### Data Collection Instruments and Data Processing

Narrative episodic joint couple interviews were conducted as part of this study. A deliberate decision was made to engage in dialogue with the spouses collectively, as this approach reflects the shared reality and facilitates negotiation processes between the individuals. Our research will deliberately consider the role of power dynamics within the interview setting, with a particular focus on the use of interruptions, attributions, accusations, and consents in relation to the discussed issues. This will be done in order to explicate the power structures and roles within the relationship and to analyze them in the context of the rehabilitation process. The couple’s interviews will be recorded using an audio recording device. To meet the criterion of intersubjective traceability, the researcher will meticulously maintain posttranscripts and research diaries, which will document not only the content of the interview but also the researcher’s personal feelings and reflections after each interview. This process will include active reflection on the researcher’s role in the field, ensuring a transparent examination of potential biases and interactions.

The project uses the transcription system “Talk in Qualitative Research” for data processing, which was developed within the framework of the documentary method [24,25].

### Data Analysis

**Figure 1:**
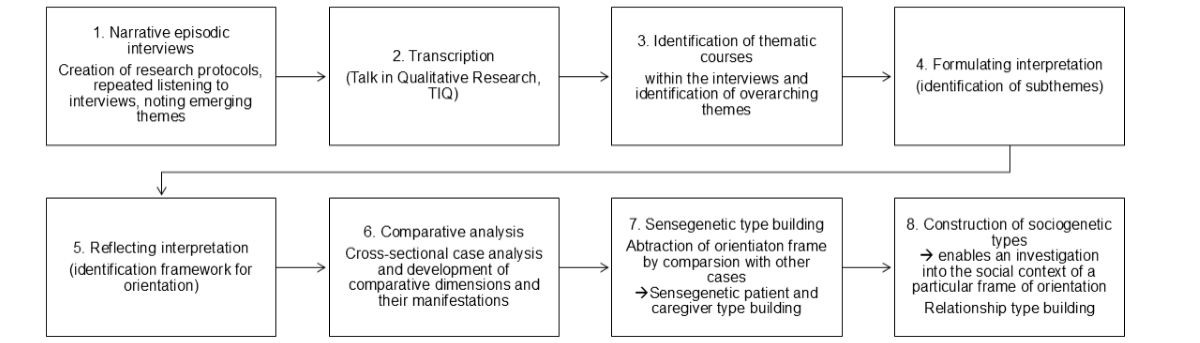
Methodical approach according to the documentary method.

The analysis of the narrative couple interviews is carried out according to Bohnsack’s documentary method (Figure 1) [24]. The main researcher will systematically engage with the material, using a step-by-step immersion process to gradually extract insights and develop comparative horizons through case comparisons. This approach will ensure a thorough exploration of each case before proceeding to the next. The material and interpretations will be discussed with the research team using a 4-eyes principle and in workshops to gain diverse perspectives, validate findings, and enhance analytical rigor.

Accordingly, the documentary method does not only remain at the level of an actor’s reflexive knowledge but also allows access to practice-relevant action. This is pertinent in relation to the attitudes and behavior of those affected and their caregiving relatives. By examining dyadic relationships retrospectively, the research seeks to understand how the meanings and dynamics within these relationships have developed and changed over time. The formulating textual interpretation summarizes what has already been interpreted by the actors in the research field. This forms the basis for the reflexive interpretation, which consists of a formal and a semantic interpretation.

### Comparative Analysis and Type Formation

The reconstruction of the habitus based on biographical narratives is conducted with the objective of determining the extent to which the habitus interpretations developed from the initial sequence can be confirmed, differentiated, or expanded through the analysis of subsequent sequences of this case. This is achieved through a comparative contrast with thematically similar sequences from other cases [26]. In a comparative analysis, the horizons of the respective cases within this study (eg, attitudes of patients and caregiving relatives) are compared and related to each other [25]. In sense-genetic type formation, we work out different orientation frameworks for dealing with a problem, for example, coping strategies for illness, and typify them [26]. Building on this, sociogenetic typing includes social contexts within the partnership and the relationship with health care professionals (Figure 1 [24]).

**Figure 2:**
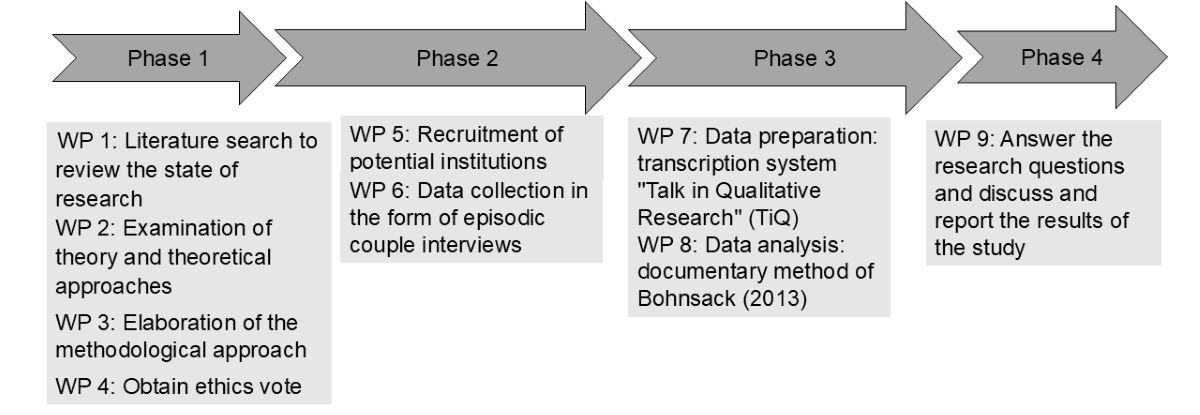
Flowchart of study procedures and work packages, own illustration. WP: work packages.

A flowchart of the study procedure and work packages is provided in Figure 2.

### Ethical Considerations

The study was approved by the ethics committee of the German Society for Nursing Science (EK-23-018) and it complies with the Declaration of Helsinki. This study is not funded. Each participant provided both verbal and written informed consent to the first author before data collection commenced. During data collection, an initial review of the material is conducted to identify emerging themes within the interviews, enabling a deeper exploration of contrasts between them. Participants were advised that they could contact the interviewer at any time if they required further information or wished to reflect on the interview’s impact. Due to the sensitive nature of discussing intimate relationship dynamics, particularly within the context of illness and rehabilitation, strict confidentiality must be upheld. Considering the emotional sensitivity of patients and their partners, the lead researcher undertook specialized training in sensitive interviewing techniques.

### Anonymization and Data Security

We will anonymize all participant names, including their place of residence and treatment facility as well as names of workplaces and contact persons. We will manage and store the raw and analytical data (transcripts) and the code list in separate, password-protected data repositories. The data will be stored for 10 years in accordance with the data protection ordinance. No payment or reimbursement of expenses is provided for participation.

## Results

The research question has been little explored and, in accordance with qualitative research principles, remains open to new and unexpected findings. The data collection started in June 2024 based on narrative interviews and is actually running. Currently, 15 dyads have been recruited and 7 have been interviewed. The current stage of the project is in work packages 6 and 7 (Figure 2). Due to the extensive procedures involved and the depth of reconstruction anticipated, we expect a sample size of up to 20 dyads. We anticipate that the study will be completed by June 2025, with data analysis concluding in the first quarter of 2025 and publication expected in the third quarter. We assume that patients and their relatives will demonstrate heterogeneity as individuals, as well as in their interactions within the dyad, regarding certain orientations such as coping with illness, motivation for therapy, and coping strategies. Furthermore, we expect that these orientations will manifest dynamically throughout the rehabilitation process and may therefore change over time.

## Discussion

### Overview

This study aims to enhance our understanding of how the relationships of affected couples evolve over the long term and how these changes impact the rehabilitation process. We propose that the nature of the relationship dynamics between patients and their partners varies based on the individual characteristics of both the patient and the partner, significantly influencing the rehabilitation experience. These dynamics may affect how patients and their partners interact and adapt to the challenges of rehabilitation. Therefore, it is essential that intervention strategies are customized to the specific types of relationships in order to provide effective therapy.

From a biopsychosocial perspective, it is crucial to involve not only the affected persons themselves but also their social environment in therapy and rehabilitation [27]. The impact of a neurological impairment extends far beyond the individual and touches on the family members and friends. Incorporating health care professionals into the everyday lives of individuals with long-term illnesses signifies a significant shift for both the patients and their partners. Frequent interactions with health care providers, whether through medical appointments or therapies, have a direct effect on patients’ health, their level of independence, and the dynamics of their shared lives.

A systematic review conducted by Weitkamp et al [28] suggests that couples dealing with chronic illness benefit from effective stress communication, supportive dyadic coping, and shared appraisals of their situation. We hypothesize that similar strategies will be identified in our study, as couples may use comparable communication techniques to manage stress and enhance mutual support. Furthermore, we expect to gain additional insights into coping strategies, including various communication patterns, and how these influence both the couple’s relationship and the rehabilitation process. This variability in coping strategies underscores the intricate ways in which couples adapt to chronic illness. We anticipate that these findings will have implications for therapeutic approaches, emphasizing the importance of recognizing and supporting diverse communication styles and options for action.

### Strengths and Limitations

The reconstruction method used in this study promises a significant gain in understanding through an in-depth analysis of the unique life worlds of the dyads. This approach aligns with Flick’s [29] advocacy for narrative and reconstructive methods, which are instrumental in revealing deeper insights within qualitative data.

Through nationwide recruitment, we aim to minimize one-sided outcomes and regional disparities in care, thereby enhancing the representativeness of our findings. The study’s methodological strengths, bolstered by the application of the documentary method, provide a solid framework for analysis. However, we acknowledge certain limitations, such as the challenges in recruiting same-sex couples and the difficulty in reaching individuals from lower socioeconomic backgrounds. Despite these challenges, we will strive to assemble a heterogeneous sample to increase the study’s validity and ensure representation across various societal strata.

### Theoretical and Practical Implications

The findings of our study are expected to contribute to the theoretical understanding of couple dynamics in the context of chronic illness and rehabilitation. We hypothesize that the variations in coping strategies will not only reflect different communication patterns but will also emerge from the specific characteristics of patients, their relatives, and the types of relationships involved. This differentiation will enhance our understanding of how various relational dynamics influence the rehabilitation process, thereby supporting existing theories while potentially introducing new dimensions to the discussion. By identifying these nuances, we aim to provide a more comprehensive framework for future research and interventions in this field. We assume that our study will support the principles of RDT by indicating that couples oscillate between change and stability, engaging in a dynamic negotiation process as they adapt to evolving relational and emotional landscapes. The theoretical grounding provided by our engagement with these theories (SI and RDT) offers a robust foundation, allowing for connections to be made with established assumptions in the field.

Enabling a deeper understanding of the interactions between professionals, patients, and their family caregivers could ultimately contribute to an increase in the quality of life of patients and their caregivers. This holistic approach has the potential to increase the effectiveness of rehabilitation by making therapy patient- and family-centered. This could create a more positive outcome in life for all those who are involved. Costs for the health care system could be reduced through effective therapy that also takes into account the mental health of relatives. Furthermore, our results could make it easier for health care professionals to provide care and could be used to support the interaction depending on the patient and the relative type as well as the relationship type.

The results will be published in both a scientific journal and a practice-oriented publication in neurological long-term rehabilitation, reaching a broad readership within the health care sector. This includes health care professionals who support affected individuals and their relatives during various phases of rehabilitation, such as doctors, nurses, physiotherapists, occupational therapists, and speech therapists. A brief summary of the results will also be prepared in patient-friendly language, similar to a patient guideline, and sent to participants upon request.

Due to a further increase in chronic diseases in the coming decades, for example, as a result of environmental, climate, and demographic changes, it is necessary to develop interventions on a physical, psychological, and social level to reduce the burden of chronic diseases [30].

### Conclusion

The successful completion of the study will provide recommendations for action for practitioners, and, if possible, will facilitate the development of a treatment concept to improve the care of people with neurological disorders and their caring relatives. A deeper understanding of psychosocial processes in connection with chronic illnesses is of great importance for the development of comprehensive, personalized care strategies.

## Abbreviations

RDT: Relational Dialectics Theory
SI: symbolic interactionism

## Data Availability

The datasets generated during this study are not publicly available due to confidentiality and ethical considerations but are available from the corresponding author on reasonable request.
